# Soybean lecithin stabilizes disulfiram nanosuspensions with a high drug-loading content: remarkably improved antitumor efficacy

**DOI:** 10.1186/s12951-019-0565-0

**Published:** 2020-01-06

**Authors:** Haowen Li, Biao Liu, Hui Ao, Jingxin Fu, Yian Wang, Yue Feng, Yifei Guo, Xiangtao Wang

**Affiliations:** 1 Institute of Medicinal Plant Development, Chinese Academy of Medical Sciences & Peking Union Medical College, No. 151, Malianwa North Road, Haidian District, Beijing, People’s Republic of China; 2 Harbin University of Commerce China, Research Center ON Life Sciences and Environmental Sciences, Harbin, 150076 People’s Republic of China; 3 Guangdong Jiabo Pharmaceutical CO., LTD, Jianshe 3rd Road, Hi-tech Industrial Development Zone, Qingyuan, 511517 Guangdong People’s Republic of China

**Keywords:** Disulfiram, Soybean lecithin (SPC), Nanosuspensions, 4T1 cells, Breast cancer

## Abstract

Disulfiram (DSF) has been considered as “Repurposing drug” in cancer therapy in recent years based on its good antitumor efficacy. DSF is traditionally used as an oral drug in the treatment of alcoholism. To overcome its rapid degradation and instability, DSF nanosuspensions (DSF/SPC-NSps) were prepared using soybean lecithin (SPC) as a stabilizer of high drug-loaded content (44.36 ± 1.09%). Comprehensive characterization of the nanosuspensions was performed, and cell cytotoxicity, in vivo antitumor efficacy and biodistribution were studied. DSF/SPC-NSps, having a spherical appearance with particle size of 155 nm, could remain very stable in different physiological media, and sustained release. The in vitro MTT assay indicated that the cytotoxicity of DSF/SPC-NSps was enhanced remarkably compared to free DSF against the 4T1 cell line. The IC_50_ value decreased by 11-fold (1.23 vs. 13.93 μg/mL, p < 0.01). DSF/SPC-NSps groups administered via intravenous injections exhibited better antitumor efficacy compared to the commercial paclitaxel injection (PTX injection) and had a dose-dependent effect in vivo. Notably, DSF/SPC-NSps exhibited similar antitumor activity following oral administration as PTX administration via injection into a vein. These results suggest that the prepared nanosuspensions can be used as a stable delivery vehicle for disulfiram, which has potential application in breast cancer chemotherapy.

## Background

**Fig. 1:**
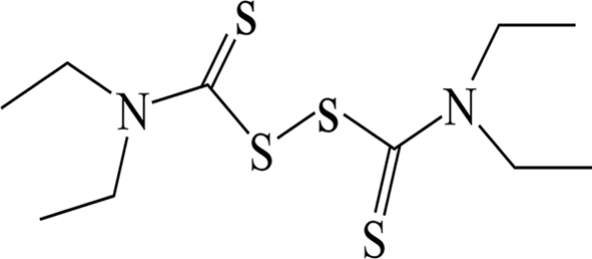
Structure of DSF

Disulfiram (DSF, Antabus, Fig. 1) is a Food and Drug Administration (FDA) approved anti-alcoholic drug since the 1940s [1, 2]. Recent researches demonstrated that DSF exhibited strong anticancer activity toward various tumor types, including breast carcinoma, liver carcinoma, colorectal cancer, non-small cell lung cancer and glioblastoma carcinoma [3–9]. DSF also inhibited tumors caused by some carcinogens [10]. DSF is an aldehyde dehydrogenase (ALDH) inhibitor. Several mechanisms of disulfiram cytotoxicity were reported, including irreversible inhibition of aldehyde dehydrogenase (ALDH) and the P-glycoprotein (P-gp) multidrug efflux pump [11, 12], superoxide dismutase (SOD) [13, 14] and activating nuclear factor-κB (NF-κB) [15, 16]. The ability of DSF to chelate divalent cations, such as copper and zinc, also improves its biological activity against tumors [17, 18]. Copper plays a critical part in oxidation–reduction reactions and the generation of reactive oxygen species (ROS) in both tumor and normal cell. The concentration of copper ions chelated DSF in tumor tissues is approximately 10 times higher than normal tissues [19]. DSF exhibited high antitumor efficacy via the formation of a complex chelate with copper ion [17, 18, 20]. For example, DSF/Cu selectively killed human breast cancer cell MDA-MB-231 and lymphocytic leukemia cells in the blood system, but it produced little impact on MCF-10A, normal breast epithelial cells, and normal lymphocytes [11, 21–23]. The higher copper-chelated DSF concentrations in tumor cells enable DSF to target cancer specifically instead of normal tissues [24]. These studies demonstrate that DSF may be used as a potent anticancer agent in clinics due to its high activity and safety.

Although DSF exhibits significant antitumor activity, it is greatly unstable in acidic gastric environment. DSF decomposes rapidly into carbon disulphide and diethylamine (DEA) [25, 26]. DSF is rapidly degraded in the blood stream by glutathione reductase, with a half-life of 4 min [14, 27]. This instability hindered the application of DSF in the clinic. Recent studies on DSF delivery systems were primarily focused on DSF-loaded nanoparticles using mPEG-PCL or PEG-PLGA as nanocarriers [5, 6, 28–30]. For example, Folate-receptor-targeted PLGA-PEG nanoparticles of disulfiram with good encapsulation efficiency (59.62%) were developed to delivery more disulfiram into breast cancer cells [5]. Tang’ team improved the plasma stability and antitumor effect in vivo of DSF by mPEG5k-b-PLGA2k/PCL3.4 k mixed micelles as carriers [6]. DSF encapsulated PLGA nanoparticles were prepared by Shahab Faghihi’ team with PLGA protecting DSF degradation and improving its cytotoxicity on MCF-7 cells. The IC50 value decreased by 2.5-fold compared to free DSF [28]. DSF-loaded porous PLGA microparticles were successfully prepared by Wang et al. with aerodynamic diameter (8.31 µm), good drug loading and antitumor efficiency using non-small-cell lung cancer A549 as a model [29]. Zhuo et al. developed an injectable DSF-NPs using mPEG_5000_ -PCL_5000_ which improved the stability of disulfiram and enhanced the DSF concentration in the blood [30]. Hanumantha Rao Madala et al. prepared passively targeted DSF-NPs which were highly stable with a size of ~ 70 nm with a > 90% entrapment. The DSF-NPs can offer a sustained drug supply for brain cancer treatment through an enhanced permeability retention [31]. Nanoparticles improve DSF stability, protect it from rapid degradation from the in vivo environment, and increase its accumulation in tumor sites [32–36]. However, the drug-loading content of these delivery systems is unsatisfactory (almost < 10%), and a large number of nanomaterials are necessarily used. An overdose of nanomaterials used as pharmaceutical excipients violate the safety standards of the FDA and may lead to serious side effects.

The present study prepared novel DSF nanosuspensions with high drug-loading content using safe nanocarriers. Soybean lecithin (SPC) is a basic substance of life, and it is a safe natural mixture of phospholipids [37]. SPC is crucial in maintain the physiological activity of biofilms and metabolism in the body [38]. SPC exhibits an amphiphilic structure [39], which may be used as an inactive ingredient to form novel formulations, and the FDA approved this use. SPC is extensively applicated in nanoscale drug delivery systems in recent years [40]. Hong C et al. developed SPC-stabilized myricetin nanosuspensions, which greatly enhanced the solubility and in vitro dissolution of myricetin and provided a myricetin formulation with a 2.57-times increased oral bioavailability [41]. Azithromycin nanosuspensions stabilized by SPC were prepared which particle size was 200 nm and were stable at room temperature after 150 days. The saturated solubility and dissolution rate of the nanosuspensions increased significantly compared to azithromycin [42]. SPC was also used as stabilizer of Annonaceous acetogenins nanosuspensions (ACGs) together with another amphiphilic polymer in our previous study. This kind of ACG nanosuspension exhibited a particle size less than 150 nm with a high drug payload (40–50%). It significantly increased the solubility of ACGs and its cytotoxicity against different tumor cells [43, 44].

The present study prepared DSF nanosuspensions (DSF/SPC-NSps) using SPC as stabilizer, which showed a high drug-loading content of approximately 44.36 ± 1.09%. The physiochemical properties of the nanoparticles, antitumor efficacy, and biodistribution were also evaluated. The results showed that DSF/SPC-NSps exhibited enhanced stability and good antitumor activity in oral and injectable formulations.

## Materials and methods

### Materials

SPC was provided by Shenyang Tianfeng Biopharmaceutical Co. Ltd. (SY-SI-170401, China). DSF was provided by Dalian Meilun Biotechnology Co. Ltd. (D1126A, China). 3-(4, 5-dimethylthiazol-2-yl)-2, 5-diphenyltetrazolium bromide (MTT) was obtained from Sigma-Aldrich Co. (St. Louis, MO, USA). PTX injection was provided by Beijing Union Pharm Ltd (China). 1, 1′-dioctadecyltetramethyl indotricarbocyanine iodide (DiR) was obtained from AAT Bioquest, Inc. (USA). All chemical reagents were analytical pure. Deionized water was used.

The 4T1 (breast cancer) cell lines were obtained from a Chinese infrastructure of cell line resource. The cells were cultured in Roswell Park Memorial Institute 1640 medium (RPMI 1640, HyClone) with 10% content of fetal bovine serum (FBS, Gibco, USA) and 100 U/mL streptomycin as well as penicillin (Gibco, USA) with 5% CO_2_ at 37 °C. Female BALB/c mice (20 ± 2 g) were provided by Beijing Huafukang Biotechnology Co., Ltd. (SPF grade). The experiment animals were provided ad libitum feeding and were acclimated for at least 7 days before experimentation.

### Preparation of DSF/SPC-NSps

DSF/SPC-NSps were prepared using an anti-solvent precipitation method [44]. 2.5 mg and 5 mg of DSF were dissolved in 0.2 mL of acetone, respectively. 5 mg of SPC was dissolved in 0.2 mL of ethanol. Next, these two solvents were mixed together, and the mixture solution was slowly injected into deionized water followed by ultra-sonication at 250 W at room temperature for 10 min. The organic solvent was removed under vacuum at 40 °C, and the DSF/SPC-NSps were obtained.

DiR is one liposoluble, fluorescent dye that can be detected under near-infrared ray. To visualize DSF biodistribution in vivo, DiR was swallowed in the hydrophobic core of DSF/SPC-NSps. DiR was dissolved in acetone together with DSF (DiR:DSF = 1:40, weight ratio). Then DiR-loaded DSF/SPC-NSps were prepared as mentioned above.

### Physicochemical characterization of DSF/SPC-NSps

The average particle size, polydispersity index (PDI) and zeta potential of DSF/SPC-NSps were determined by dynamic light-scattering method (DLS, Zetasizer Nano ZS; Malvern Instruments, UK) at 25 °C. Each sample was measured three times with 14 scans.

6 µL of water-diluted sample (100 µg/mL) was dropped on a 300-mesh copper sheet, air-dried and colored by 20 µL of 2% (w/v) uranyl acetate for 30 s. Then the morphology of DSF/SPC-NSps were observed under transmission electron microscopy (TEM; JEOLLtd, Japan) at an accelerating voltage of 120 kV.

### Differential scanning calorimetry (DSC) characterization

DSC thermal profile was obtained using a differential scanning calorimeter (DSC, Q200, TA Instruments, DE). 5 mg of powder sample (SPC, DSF powder, DSF/SPC-NSps lyophilized powder, and DSF powder with SPC physical mixture) sealed in standard aluminum pan was detected from 0 to 110 °C (10 °C/min, nitrogen atmosphere).

### X-ray diffraction (XRD) measurements

XRD patterns of powder sample (SPC, DSF powder, DSF/SPC-NSps lyophilized powder, and DSF powder with SPC physical mixture) were measured by X-ray diffractometer (DX-2700, China) with Cu-Kα radiation generated at 100 mA and 40 kV. Samples were scanned over an angular range of 3–80° of 2θ, with a step size of 0.02° and a count time of 3 s per step.

### HPLC determination of DSF

The DSF concentration of DSF/SPC-NSps were measured in an HPLC system (DIONEX Ultimate 3000, USA). A Symmetry C18 column (250 mm × 4.6 mm, 5 μm, Venusil XBP) was used at 25 °C for chromatographic separation. The mobile phase was constituted of water and acetonitrile (30/70, v/v). The flow rate was 1 mL/min. The detection wavelength was 210 nm (UV detector, DIONEX).

### Stability of DSF/SPC-NSps in various physiological solutions

DSF/SPC-NSps were incubated with 0.9% NaCl, 5% glucose (1:1, v/v), PBS (pH 7.4), artificial gastric and intestinal fluid (1:4, v/v) at 37 °C. One milliliter of the mixture was removed. The sizes and particle distribution were analyzed using DLS, and the concentration of DSF was determined using HPLC at specific time intervals. Each sample was performed in triplicate.

### Stability of DSF/SPC-NSps in rat plasma

In vitro plasma stability tests were performed as follows. The mixture solution of rat plasma and DSF/SPC-NSps (1 mg/mL, 1:4, v/v) was incubated at 37 °C. 1 mL of the mixture solution was taken out and measured for particle size changes at specific time intervals. The concentration changes of DSF were determined using HPLC. The concentration changes of DSF incubated with RPMI 1640 medium were also determined for the next MTT assy. Each experiment was performed in triplicate.

### Drug loading and release behavior of DSF/SPC-NSp**s**

Lyophilized DSF/SPC-NSps were dissolved in acetonitrile. The content of DSF was determined by HPLC. The drug loading content (DLC) of DSF/SPC-NSps was calculated as follows:

$${\text{DLC }}\left( \% \right) \, = {\text{ V}}*{\text{C}}/{\text{W}} \times 100\%$$

(V: acetonitrile volume; C: DSF concentration; W: lyophilized powder of DSF/SPC-NSps weight)

The in vitro behavior of drug release of DSF/SPC-NSps was performed as follows. PBS with 1% polysorbate 80 (0.01 M, pH 7.2–7.4) was chosen as dissolution medium. DSF suspensions (1 mg/mL, dispersed in 10 mL of PBS containing 1% polysorbate 80) and DSF/SPC-NSps (2 mL, 1 mg/mL, dispersed in containing 1% polysorbate 80) were encapsulated in dialysis tubes (MWCO: 8000–14,000, Sigma). The dialysis tubes were dipped in 50 mL of PBS containing 1% polysorbate 80 (0.01 M, pH 7.2–7.4) with continuous stirring at 150 rpm at 37 °C. One milliliter of the external liquid was withdrawn at each time intervals, and then the system was supplemented with the same volume of newly-prepared dissolution medium. The dissolution medium was renewed every 24 h. The cumulative release profile of DSF/SPC-NSps was calculated according to the increase of DSF in the external release medium. The above experiments were performed in triplicate.

### MTT assay

In vitro cell cytotoxicity of DSF/SPC-NSps was evaluated via the MTT assay. 4T1 cells (1.0 × 10^4^ cells/well in 150 μL) were sowed in 96-well plates and incubated 24 h in 5% CO_2_ at 37 °C. DSF/SPC-NSps with different concentrations and free DSF solution (DSF in DMSO) were added and incubated for 48 h. Then 20 μL of the MTT solution (5 mg/mL in PBS) were added into each well and incubated for 4 h. The medium was removed and 150 μL of DMSO was added to dissolve the formazan crystals. The maximum absorbance was measured by an ELISA plate reader at 570 nm (Biotek, USA). The cell viability rate was calculated as follows:

$${\text{Cell viability rate }}\left( \% \right) \, = {\text{ OD}}_{\text{t}} /{\text{OD}}_{\text{c}} 100\%$$

(OD_t_: the mean optical density (OD) of the tested group; OD_c_: the mean OD of the control group. Each group’s half-inhibitory concentration (IC_50_) value was calculated by GraphPad Prism software, Version 7 (GraphPad Software, Inc., USA).

### In vivo antitumor efficacy and biodistribution study

Female Balb/c mice (20 g ± 2 g) were inoculated with 4T1 cells (8.0 × 10^5^ cells each mouse) subcutaneously in the right armpit. The 4T1 tumor-bearing mice were divided into seven groups randomly (8 mice per group) when the tumor volume came up to 100 mm^3^. Mice were administered normal saline solution (NS, negative control group), PTX injection (positive control group, 8 mg/kg), and DSF/SPC-NSPs at concentrations of 5, 10 and 20 mg/kg in a final volume of 0.2 mL via the tail vein. Another two groups were administered a free DSF solution (20 mg/kg) or DSF/SPC-NSps (20 mg/kg) via gavage. The intravenous injection (*i.v.*) groups were injected through tail vein every 2 days, and the oral groups (*i.g.*) received gavage daily. All groups were administered continuously for 14 days. Tumor volume and body weight were recorded during the whole experiment.

DiR was loaded in the DSF/SPC-NSps group (20 mg/kg, *i.v.*) during the last administration (DSF/DiR weight ratio of 40/1) via intravenous injection. All the mice were sacrificed 24 h later, and organs (tumor, heart, liver, lung, spleen and kidney) were excised and imaged using a Living Image software (Version 4.2) for quantitative analyses. In vivo fluorescence was imaged using IVIS Living Image ^®^ 4.4 (Caliper Life Sciences, USA).

All other groups were sacrificed via cervical spine dislocation. The complete tumor of each mouse was dissected. The tumor was weighed, and the tumor inhibition rate (TIR %) was calculated according to the formula:

$${\text{TIR}}\% \, = \, \left( { 1- {\text{W}}_{\text{t}} /{\text{W}}_{\text{n}} } \right) \, \times 100\%$$

(W_n_: the average tumor weight of NS group; W_t_ : the average tumor weight of the experimental group)

### Statistic analysis

Statistic analysis between experimental groups was calculated by independent-samples *T* test (two groups) and one-way analysis variance (F-test for more than two groups) in IBM SPSS Statistics software, Version 21 (IBM Corporation, USA). P < 0.05 was considered as statistically significant.

## Results and discussion

### Preparation and characterization of DSF/SPC-NSps

**Fig. 2:**
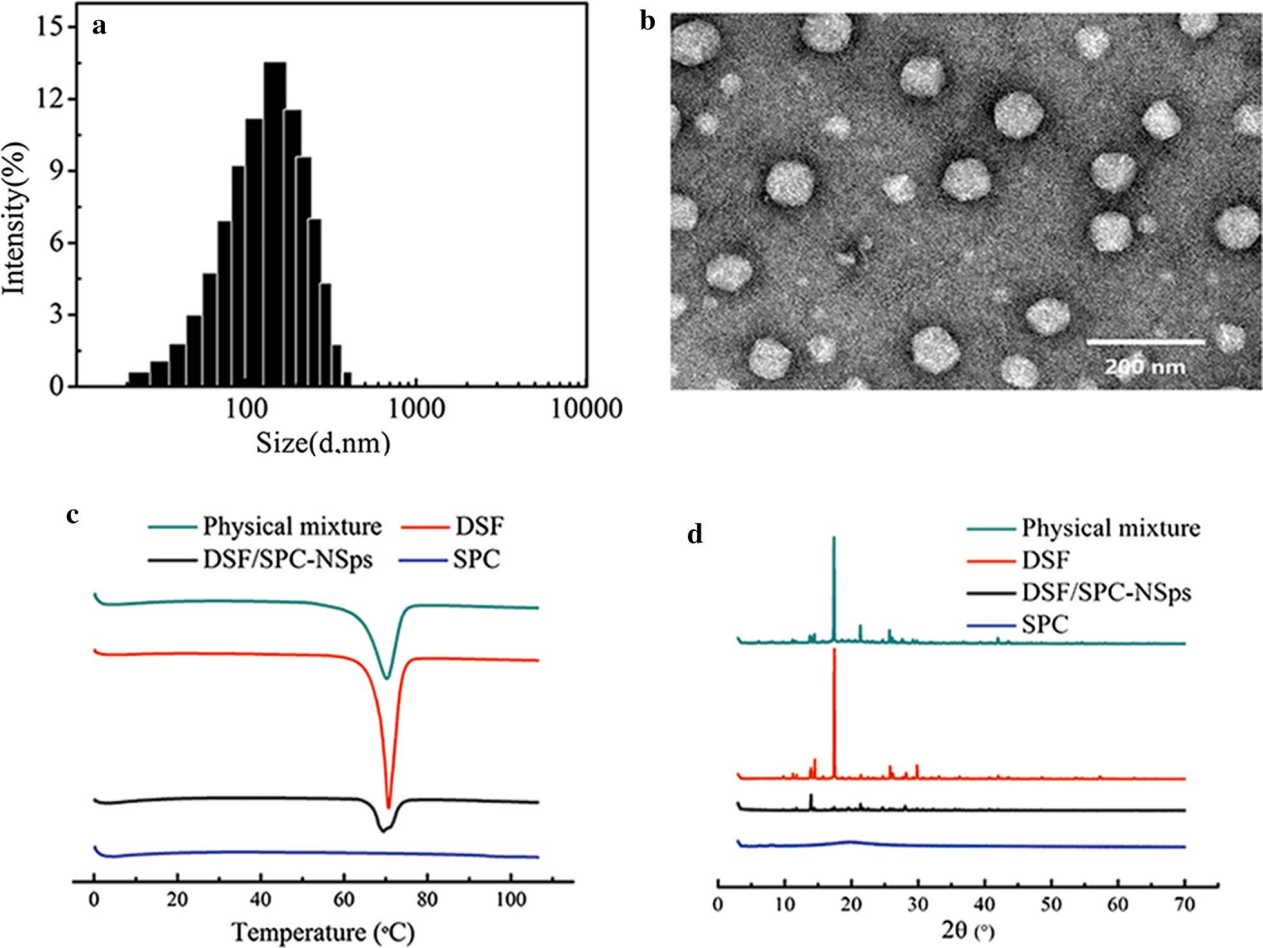
Characterization of DSF/SPC-NSps. **a** Particle size distribution of DSF/SPC-NSps. **b** Transmission electron microscopy images of DSF/SPC-NSps. **c** Differential scanning calorimetry thermograms of DSF bulk powder, SPC, DSF/SPC-NSps, and the physical mixture of DSF bulk powder and SPC. **d** XRD patterns of DSF bulk powder, SPC, DSF/SPC-NSps, and the physical mixture of DSF bulk powder and SPC

Two different weight ratios of DSF and SPC were successfully prepared at the drug/carrier ratios of 1:2 and 1:1. DLS measurement revealed that the particle size of DSF/SPC-NSps was decreased and the drug-loading content was increased with the change in the drug/carrier ratio from 1:2 to 1:1 (Table 1). The DSF/SPC-NSps exhibited a good particle size of 155 nm when the drug/carrier ratio was 1:1, with a narrow particle size distribution (PDI = 0.22) (Fig. 2a), and the drug-loading content was 44%. In comparison, the size of DSF suspended in water was too large to be determined using DLS. Due to the desirable particle size and drug-loading content, the DSF/SPC weight ratio of 1:1 was chosen as the optimizing formula for DSF/SPC-NSps preparation in the succeeding research.

**Table 1:** Size, PDI and zeta potential of the resultant DSF/SPC-NSps at different ratios with a final drug concentration of 1 mg/mL (mean ± SD, n = 3)

Ratios of DSF and SPC	Size (nm)	PDI	Zeta (mV)	Drug-loading rate
1:2	219.72 ± 5.69	0.21 ± 0.01	− 22.10	23.77 ± 2.13%
1:1	155.50 ± 5.17	0.22 ± 0.02	− 17.83	44.36 ± 1.09%

TEM observation revealed that DSF/SPC-NSps were spherical and evenly distributed (Fig. 2b). The size was 84.4 ± 5.8 nm, a little smaller than that determined by DLS because DLS measures the equivalent radius of the particle size in pure water with the hydration layer. DSF/SPC-NSps exhibited a small size of < 200 nm, which may be easily delivered to the tumor site via the EPR effect [45].

### Differential scanning calorimetry and X-ray diffraction characterization

The DSC investigation displayed that both DSF powder and physical mixture showed an acute endothermic peak at around 70 °C (Fig. 2c), which was the melting point of DSF. The DSF/SPC-NSps showed a relatively weak endothermic peak in the identical location, which suggested that a crystalline form of DSF existed in the DSF/SPC-NSps.

The XRD patterns of the DSF powder, SPC, physical mixture of DSF powder with SPC and DSF/SPC-NSps were tested under the same conditions (Fig. 2d). The diffractograms of DSF powder and physical mixture showed acute diffraction peaks of crystallinity, which indicated that DSF existed primarily in crystalline form in these two systems. The lyophilized DSF-NSps exhibited similar diffraction peaks to those of DSF bulk powder and the physical mixture, with the exception of much lower signal intensity. This indicated that DSF was also in a crystalline form in the DSF/SPC-NSps. The disproportionate signal intensity reduction of DSF/SPC-NSps at 19° suggested that the crystalline form of DSF in DSF/SPC-NSps may not be totally identical to that of free DSF. The results of DSC and XRD suggested that DSF in nanosuspensions primarily exist in the form of a crystal.

### Stability of DSF/SPC-NSps in physiological media

**Fig. 3:**
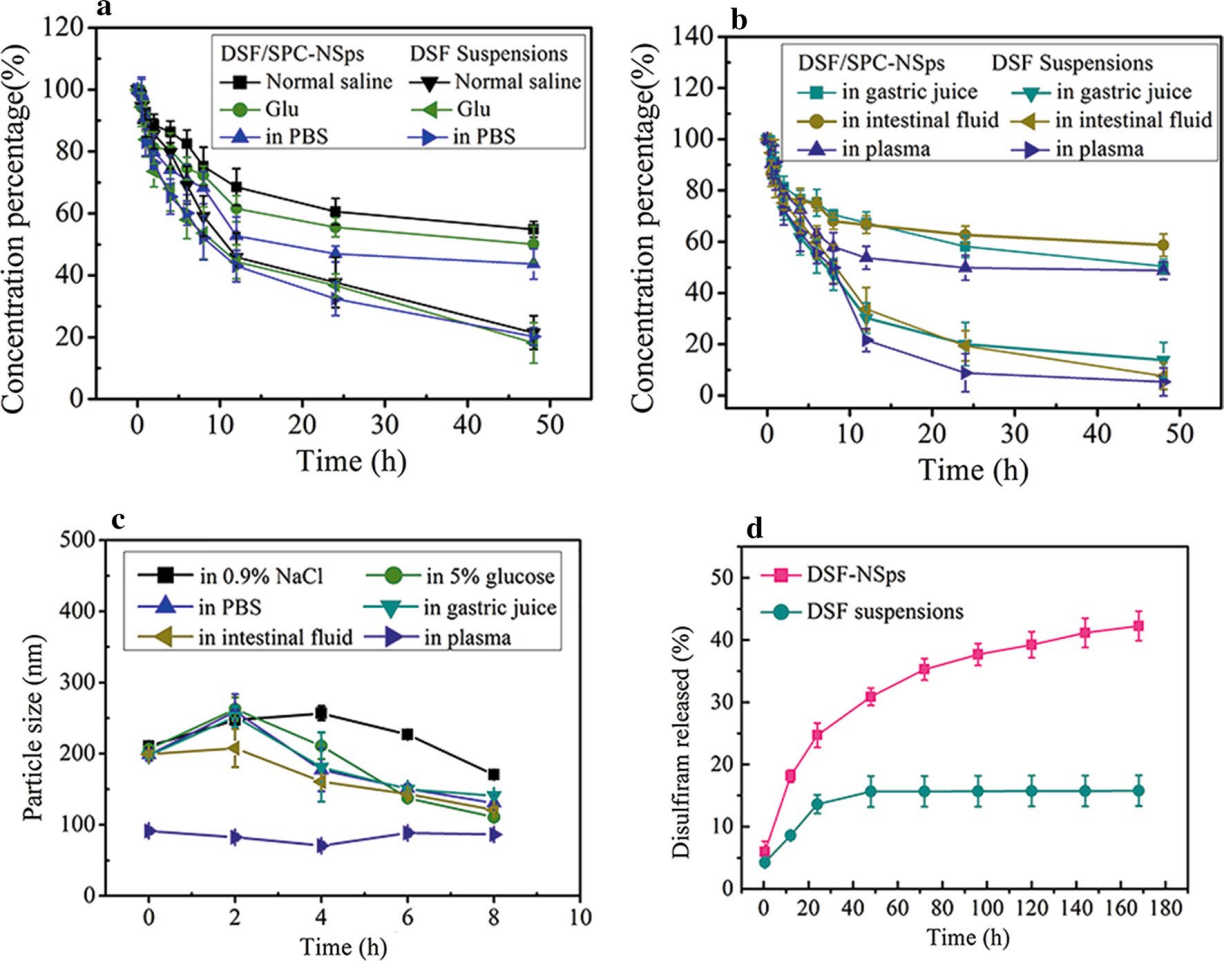
**a** DSF content change curves of DSF/SPC-NSps in 0.9% NaCl, 5% glucose and PBS at 37 °C. **b** DSF content change curves of DSF/SPC-NSps in artificial gastric juice, artificial intestinal fluid, and plasma at 37 °C. **c** Particle size change curves of DSF/SPC-NSps in 0.9% NaCl, 5% glucose PBS, artificial gastric juice, artificial intestinal fluid, and plasma at 37 °C. **d** In vitro cumulative release profiles of DSF from DSF/SPC-NSps at 37 °C. All data are presented as the mean ± SD (n = 3)

Figure 3c shows the particle size changes of DSF/SPC-NSps in different physiological media. DSF/SPC-NSps were stable for 8 h in normal saline, 5% glucose solution, PBS, artificial gastric juice, artificial intestinal fluid, and plasma. The aggregation phenomenon did not occur during the incubation procedure. Notably, the size of DSF/SPC-NSps grew a bit larger after the particles were suddenly mixed with different physiological media because its surface properties changed. However, the system regained stability 2 h later because no obvious changes in particle size were observed, and PDI was relatively stable (Fig. 3c). These results indicated that the DSF/SPC-NSps structure is stable in various media. The DSF concentrations of DSF/SPC-NSps and DSF water suspensions were determined using HPLC at the same time interval until 48 h (shown in Fig. 3a, b). A total of 50–60% of DSF remained in DSF/SPC-NSps until 48 h. In comparison, the content of DSF remaining in water suspensions was less than 20%, which was consistent with previous report about DSF degradation of DSF in aqueous solution [46]. This result also demonstrated that DSF/SPC-NSps possessed much higher stability than DSF suspensions in vitro.

### Drug-release behavior of DSF/SPC-NSps

There was a burst release of DSF/SPC-NSps within 24 h with a cumulative drug release that reached approximately 25%, followed by a sustained and steady cumulative drug release that reached 42.26 ± 2.35% until 168 h (Fig. 3d). DSF suspensions were used as the control group and treated under identical conditions. The cumulative drug release of DSF water suspensions was only 15.60% after 48 h and remained stagnant until 168 h. The small size of DSF/SPC-NSps compared to DSF water suspensions increased surface area and enhanced drug solubility. Therefore, the sustained drug release was increased.

The burst release of DSF from NSps was because of the fast dispersion of the surface drug into release media. After the burst release phase, a sustained drug release profile was observed until 160 h, which provides DSF for a continuous effect over a long period. The sustained and prolonged drug release may be relevant to the drug diffusion and matrix erosion mechanisms [5, 47]. This moderate release rate avoids drug leak during circulation and guarantees sufficient drug concentrations at the tumor site.

### MTT assay

**Fig. 4:**
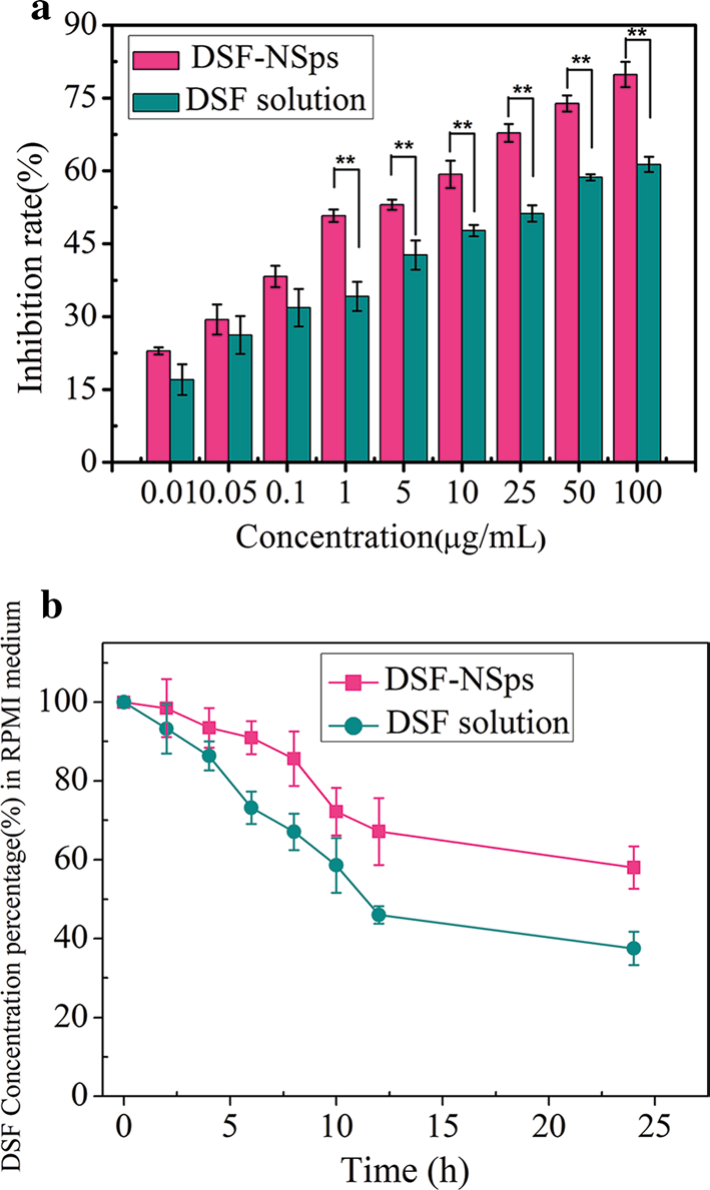
**a** In vitro antiproliferative activity of the DSF solution and DSF/SPC-NSps against 4T1 cells for 48 h using the MTT assay. **b** DSF content change curves of DSF/SPC-NSps and DMSO solution in RPMI 1640 medium 37 °C ** p < 0.01. All data are presented as the mean ± SD (n = 6)

The two DSF formulas both inhibited 4T1 tumor cell growth in a dose-dependent manner (Fig. 4a). DSF/SPC-NSps exhibited much stronger cytotoxicity against 4T1 cells than the free DSF solution at the tested concentration range from 0.01 to 100 μg/mL. The cytotoxicity of DSF/SPC-NSps was enhanced significantly compared to free disulfiram. The IC_50_ value decreased 11-fold (1.23 vs. 13.93 μg/mL, DSF/SPC-NSps vs. free disulfiram, p < 0.01). The results showed that DSF/SPC-NSps exhibited a stronger cytotoxic effect on 4T1 cells than the free DSF solution. The reasons for this phenomenon are as follows. Firstly, the free DSF solution was unstable in an aqueous medium when incubated together with tumor cells, and therefore, it was partially degraded before cellular uptake (shown in Fig. 4b). While nanosuspensions maintained most DSF molecules intact for uptake by tumor cells during MTT assay. Secondly, free DSF solution can be merely transported inside cells via passive diffusion. DSF/SPC-NSps can be uptaken into the tumor cells by endocytosis [48, 49]. Besides, the constitute of SPC was similar to cell membranes and had good bioavailability, therefore, DSF/SPC-NSps will be more easier to get into cells [38]. Thus, DSF/SPC-NSps manifest much more effective cytotoxicity than DSF solution.

### In vivo antitumor efficacy and in vivo biodistribution

**Fig. 5:**
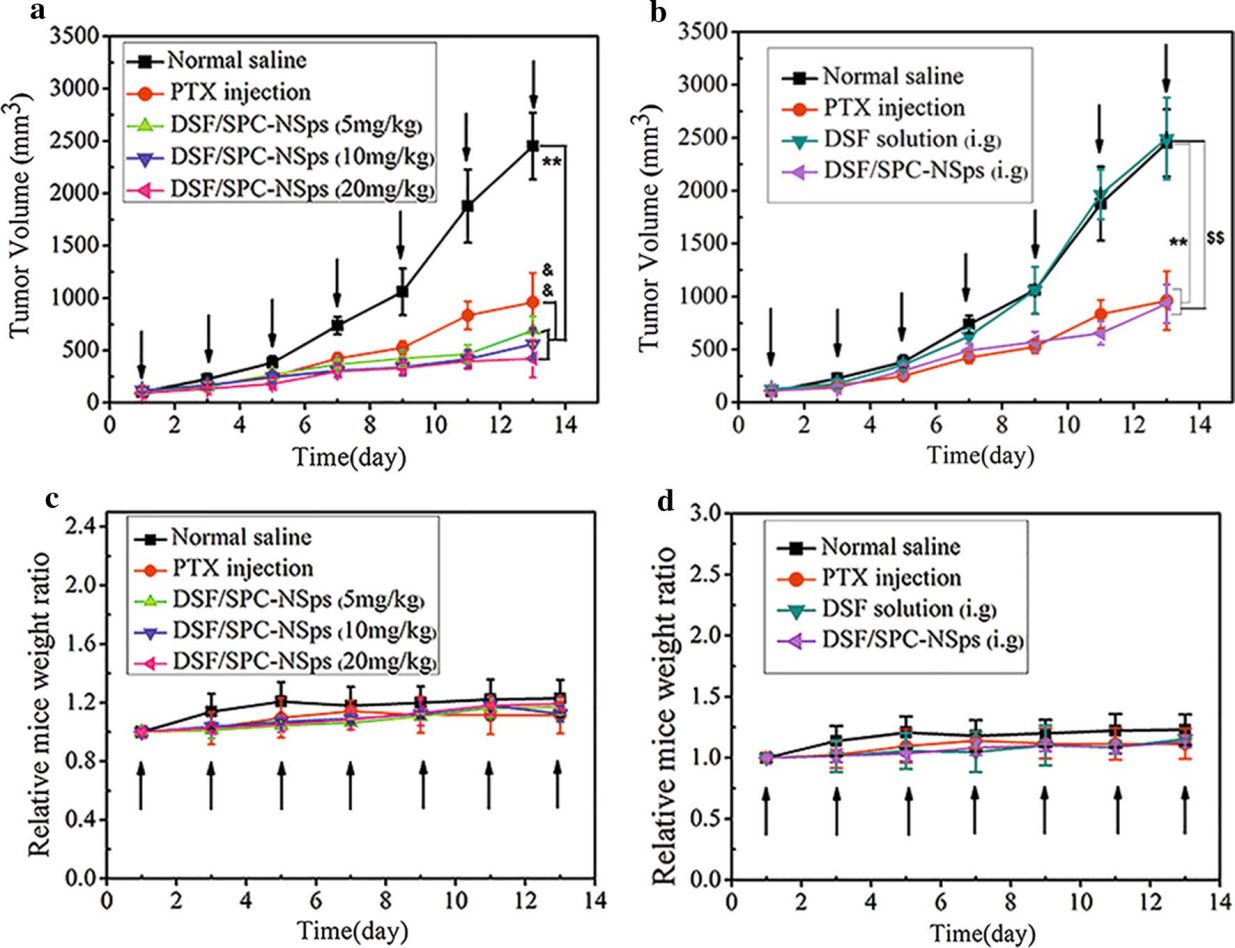
In vivo antitumor experiments in 4T1 tumor-bearing mice. **a** Growth of relative tumor volume over time. (*i.v.*) **b** Growth of relative tumor volume over time. (*i.g.*) **c** Average body weight change of mice along with time. (*i.v.*) **d** Average body weight change of mice along with time. (*i.g.*) (**p < 0.01 vs. normal saline, ^$$^p < 0.01 vs. DSF solution, ^&&^p < 0.01 vs. PTX group)

The antitumor efficacy of DSF/SPC-NSps was performed on 4T1 tumor-bearing mice using normal saline as negative control and PTX injection as positive control. The time-related tumor volume changes are shown in Fig. 5a, b. The tumor volume of negative control group increased 25-fold and came up to 2500 mm^3^. The PTX injection group exhibited moderate antitumor efficacy with a tumor volume increase of 9.8-fold. Three groups treated with DSF/SPC-NSps i.v. exhibited a dose-dependent antitumor effect, and the tumor volumes increased 6.3-, 5.2-, and 4.7-fold at 5, 10 and 20 mg/kg, respectively. DSF/SPC-NSps produced a better antitumor effect than PTX injection (8 mg/kg) at a lower dose (5 mg/kg). The oral DSF solution exhibited no anticancer effect, with a 24.3-fold increase in tumor volume. In contrast, oral DSF/SPC-NSps produced a similar antitumor effect as the PTX injection, with a 9.6-fold increase in tumor volume. A significant difference was obtained between the DSF/SPC-NSps groups and negative control group (p < 0.01). The three DSF/SPC-NSps intravenous administration groups exhibited a significant difference compared to the PTX injection group (p < 0.01).

**Fig. 6:**
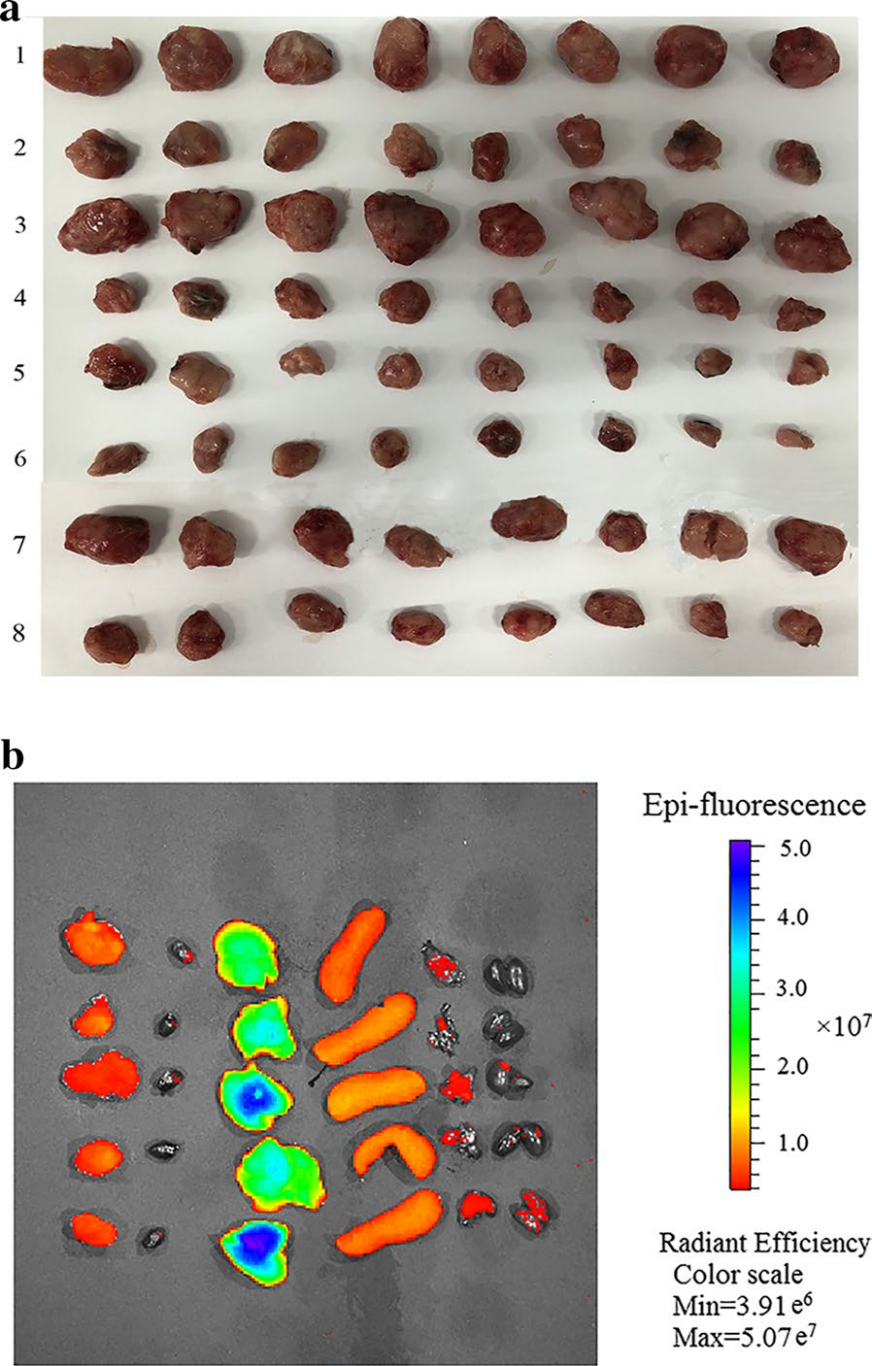
**a** Tumor images (1: saline; 2: PTX injection; 3: SPC-NSps blank *i.v.*; 4:DSF/SPC-NSps *i.v.* 5 mg/kg; 5: DSF/SPC-NSps *i.v.* 10 mg/kg; 6: DSF/SPC-NSps *i.v.* 20 mg/kg; 7: DSF solution *i.g.*; 8: DSF/SPC-NSps *i.g.*) (n = 8, mean ± SD). **b** Distribution of DiR/DSF/SPC-NSps in the major organs of tumor-bearing mice at the end of the experiment (24 h). In vivo fluorescent images of tumor tissue and other vital organs (from left to right: tumor, heart, liver, spleen, lung, and kidney) (n = 5, mean ± SD)

The tumor sizes in the DSF/SPC-NSps groups were obviously smaller than the blank control group (Fig. 6a). The tumor inhibition rates calculated by the average tumor weights are shown in Table 2. The inhibition rate relative to the saline control was 55.01% for the PTX injection group, 69.20%, 74.80% and 80.00% for 5, 10 and 20 mg/kg DSF/SPC-NSps *i.v.* groups, respectively, and 57.06% for the DSF/SPC-NSps *i.g.* group. The inhibition effects of DSF/SPC-NSps increased 1.25-, 1.35- and 1.45-fold at the dose 5, 10 (p < 0.01) and 20 mg/kg (p < 0.01), respectively, compared to the PTX injection group. Antitumor efficacy was stronger as the dose increased. The oral DSF solution did not exhibit an obvious antitumor effect due to its instability and quick degradation in gastric juice. Oral DSF/SPC-NSps showed a much better antitumor efficacy (TIR, 57.06% vs. 8.19%, p < 0.01) compared to the oral DSF solution. Oral administration of DSF/SPC-NSps achieved similar antitumor efficacy as the PTX injection (TIR, 57.06% vs. 55.01%), which may increase patient compliance compared to intravenous administration.

**Table 2:** In vivo antitumor effects of different groups against 4T1 tumors in mice

Group	Dose (mg/kg)	Spleen coefficient (%)	Liver coefficient (%)	Tumor weight (g)	Inhibition rate (%)
Saline	–	5.12 ± 0.39	3.48 ± 0.46	3.54 ± 0.55	–
DSF solution (*i.g.*)	20	5.19 ± 0.60	3.90 ± 0.47	3.25 ± 0.46	8.19
PTX Injection (*i.v.*)	8	4.64 ± 0.55	2.93 ± 0.38	1.59 ± 0.06**^##$$^	55.01
DSF/SPC-NSps (*i.g.*)	20	4.72 ± 0.70	3.02 ± 0.61	1.52 ± 0.132**^##$$^	57.06
DSF/SPC-NSps (*i.v.*)	5	6.50 ± 0.45	4.46 ± 0.29	1.09 ± 0.21**^##$$^	69.20
	10	6.06 ± 0.48	4.53 ± 0.32	0.89 ± 0.23**^##$$&&^	74.80
	20	6.18 ± 0.62	4.48 ± 0.36	0.71 ± 0.17**^##$$&&^	80.00

The results are presented as the mean ± SD, n = 8 **p < 0.01 vs. normal saline; ^&&^p < 0.01 vs. PTX injection; ^$$^p < 0.01 vs. DSF solution

To estimate the in vivo distribution of DSF, DiR was encapsulated in DSF/SPC-NSps. Figure 6b shows the fluorescence intensity in mouse organs. Most fluorescence signal emission was detected in the liver and spleen 24 h after intravenous injection of DSF/SPC-NSps, and tumor tissue also exhibited some fluorescence retention. The average percentage of tumor fluorescence degree to liver fluorescence degree in mice was 19.48 ± 5.57%, which indicates that DSF/SPC-NSps accumulated in tumor site. The strong fluorescence degree detected in the liver and kidney was due to the phagocytosis of NSps by macrophagocyte of the reticuloendothelial system (RES) [50, 51].

Systematic toxicity was evaluated using average body weight change profile (Fig. 5c, d) and the liver and spleen indexes of each group (Table 2). There were no significant differences in average body weight change for mice in each group throughout the experiment. No obvious body weight reduction was observed. The liver and spleen indexes of the experimental groups were not significantly different from the normal saline group (p > 0.05), which indicates a very low-level tendency of systemic toxicity and good biosafety of DSF/SPC-NSps.

## Conclusion

The present research successfully prepared DSF/SPC-NSps with the mean diameter of 155 nm, a narrow size distribution, and an excellent drug loading content of 44.36 ± 1.09%. DSF/SPC-NSps showed remarkable stability in various physiological media and a continuous release manner for 160 h in vitro. DSF/SPC-NSps demonstrated much stronger cytotoxicity against 4T1 cells than free DSF solution in vitro, with an 11-fold decrease in IC_50_ value. In vivo experiments demonstrated that the oral administration of DSF/SPC-NSps produced similar antitumor activity as a PTX injection, and intravenous administration exhibited much higher antitumor efficacy than the PTX injection. DSF should be a potent antitumor drug in the clinic due to its excellent effects against tumor cells, low price and good tolerance by the human body compared to other chemotherapy agents in the market. DSF/SPC-NSps solved the rapid degradation problem of DSF and exhibited excellent antitumor effects. Therefore, it may become a promising delivery system for cancer treatment.

## Abbreviations

DSF: disulfiram
SPC: soybean lecithin
PTX injection: paclitaxel injection
DSF-NSps: DSF was encapsulated into nanoparticles
DSF/SPC-NSps: DSF nanoparticles stabilized by SPC
FBS: fetal bovine serum
DiR: 1, 1′-dioctadecyltetramethyl indotricarbocyanine iodide
